# Structural Properties, Cytotoxicity, and Anti-Inflammatory Activity of Silver(I) Complexes with tris(p-tolyl)Phosphine and 5-Chloro-2-Mercaptobenzothiazole

**DOI:** 10.1155/2010/386860

**Published:** 2010-03-29

**Authors:** L. Kyros, N. Kourkoumelis, M. Kubicki, L. Male, M. B. Hursthouse, I. I. Verginadis, E. Gouma, S. Karkabounas, K. Charalabopoulos, S. K. Hadjikakou

**Affiliations:** ^1^Section of Inorganic and Analytical Chemistry, Department of Chemistry, University of Ioannina, 45110 Ioannina, Greece; ^2^Medical Physics Laboratory, Medical School, University of Ioannina, 45110 Ioannina, Greece; ^3^Department of Chemistry, A. Mickiewicz University, ul. Grunwaldzka 6, 60-780 Poznań, Poland; ^4^Department of Chemistry, University of Southampton, Highfield, Southampton SO17 1BJ, UK; ^5^Department of Experimental Physiology, Medical School, University of Ioannina, 45110 Ioannina, Greece

## Abstract

The synthesis and characterization of the silver(I) chloride complex of formula {[AgCI(CMBZT)(TPTP)_2_] · (MeOH)} (**1**) (CMBZT = 5-chloro-2-mercaptobenzothiazole, TPTP = tris(p-tolyl)phosphine) is described. Also the structure of the hydrate derivative {[AgCI(TPTP)_3_] · (0.5 · H_2_O)} (**2**) of the corresponding known anhydrous silver complex (Zartilas et al., 2009), and the polymorph **3** of the known [AgI(TPTP)_3_] complex (Zartilas et al., 2009) were determined and compared with the known ones. In addition, the structure of the known one silver(I) cluster {[AgI(TPTP)]_4_} (**4**) (Meijboom et al., 2009) was re-determined at 120(2) K and possible Ag-Ag interactions were analyzed. The compounds **1–4** were characterized by X-ray crystallography at r.t (**1**) and 120 K (**2–4**). All these complexes and {[(Et_3_NH)^+^]_2_ · [Ag_6_(*μ*_3_-Hmna)_4_(*μ*_3_-mna)_2_]^2−^ · (DMSO)_2_ · (H_2_O)} (**5**) (Hmna = 2-mercaptonicotinic acid) were evaluated for cytotoxic and anti-inflammatory activity. The *in vitro* testing of cytotoxic activity of **1–5** against leiomyosarcoma cancer cells (LMS), were evaluated with Trypan Blue and Thiazolyl Blue Tetrazolium Bromide or 3-(4.5-dimethylthiazol-2-yl)-2.5-diphenyltetrazolium bromide (MTT) assays. The flow cytometry assay for complex **1** and showed that at 15 *μ*M of **1**, 62.38% of LMS cells undergo apoptosis, while 7% of LMS cells undergo cell necrosis. The antitumor activity of **3** is comparable with that of its reported polymorph (Zartilas et al., 2009). The anti-inflammatory, activity of complexes **1–3** and **5** was also studied. The activity towards cell viability was 2 > 3 > 5 > 1 > 4, while the order of the inhibitory activity in cell growth proliferation follows the order, 2 > 3 > 1 > 4 > 5. The anti-inflammatory activity on the other hand is 1 > 2 > 5 > ⋯ >3.

## 1. Introduction

Silver(I) complexes with sulfur containing ligands exhibit a wide range of applications in medicine, in analytical chemistry and in the polymer industry [1–5]. The biomedical applications and uses of silver(I) complexes are related to their antibacterial action [6, 7] which appears to involve interaction with DNA [8]. Silver sulphadiazine is a topical anti-infective, used worldwide for dermal injuries, approved by the US Food and Drug Administration [9]. It has a broad antibacterial spectrum including virtually all microbial species likely to infect the burn wound [9]. Recently, Ag(I) complexes have also been studied for their antitumor activity [10–12]. The results showed that [AgBr(TPTP)_3_] complex possessed the strongest activity against leukemia (L1210), human T-lymphocyte (Molt4/C8 and CEM) and LMS cells [12]. The exact mechanism of the anti-tumor action of Ag(I) compounds is still unknown. It is well known, however, that many drugs which inhibit the growth of tumor cells act either by interfering with DNA bases and/or nucleotides or with the metalloenzymes that are necessary for the rapid growth of malignant cells [13, 14]. Thus, the molecular design and structural characterization of silver(I) complexes is an intriguing aspect of bioinorganic chemistry and metal-based drugs research [15, 16]. The ability of silver(I) complexes to adopt geometries with variable nuclearities and structural diversity also makes the study of silver(I) chemistry very attractive [3–5, 17]. This self-assembly process between metal ions and ligands is known to depend on steric and interactive information stored in the ligand and is governed by the metal ions through the demands of their coordination geometry [18, 19]. Recently, a new type of aromaticity, due to a cyclical delocalization of d as well as (d-p) *π*-type orbital electron density instead of the usual p orbitals on metal-ligand rings, has been reported [20–29], which may introduce greater stability to the higher-order structures. 

**Scheme 1:**
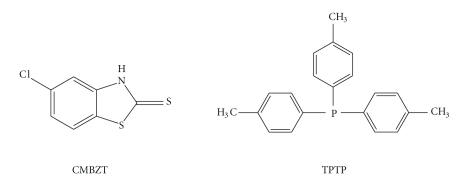


 This paper, reports the synthesis of the new mixed ligand silver(I) chloride complex with the heterocyclic thioamide 5-chloro-2-mercaptobenzothiazole (CMBZT, C_7_H_4_ClNS_2_) and tri(p-tolyl)phosphine (TPTP, C_21_H_21_P) (Scheme 1) of formula {[AgCl(CMBZT)(TPTP)_2_] · (MeOH)} (**1**). The hydrate {[AgCl(TPTP)_3_] · (0.5 · H_2_O)} (**2**) of the known anhydrous silver complex [10] and a polymorph, **3**, of the known [AgI(TPTP)_3_] complex [10, 30] were also isolated. The crystal structure of **4**  {[AgI(TPTP)]_4_} has been previously reported at 100(2) K [31] but we have extended our studies here in the determination of its quasiaromaticity, which results in strong Ag–Ag interactions and thus greater stability, using data collected at 120 K. Complexes **1**–**4** and the known silver(I) cluster {[{[(Et_3_NH)^+^]_2_ · [Ag_6_(*μ* *_3_-*Hmna)_4_(*μ* *_3_-*mna)_2_]^2-^ · (DMSO)_2_ · (H_2_O)} (**5**) (H_2_MNA = 2-mercaptonicotinic acid, C_6_H_5_NO_2_S) [32] were tested for their in vitro cytostatic activity against leiomyosarcoma cancer cells from Wistar rats. Finally, the anti-inflammatory activity of complexes **1**–**3** and **5** were also evaluated and the results correlated with those of their anti-tumor activity. 

## 2. Results and Discussion

### 2.1. General Aspects

Complex **1** was synthesized by heating at 50°C an acetonitrile/methanol solution of silver(I) chloride, TPTP and CMBZT in 1 : 2 : 1 molar ratio (reaction I):

(I) $$\begin{array}{cc} & \text{AgCl}+2\text{TPTP}+\text{CMBZT}\\ & \;\underset{50^{{}^{\circ}}\text{C}}{\overset{{\text{CH}}_{3}\text{CN}/\text{MeOH}}{\to }}\;\left[\text{AgCl}\left(\text{CMBZT}\right){\left(\text{TPTP}\right)}_{2}\left(\text{MeOH}\right)\right]\;\;\left(1\right) \end{array}$$

Complexes **2**–**4** [10, 30, 31] were synthesized by heating, under reflux, a toluene solution of silver(I) halides with TPTP in the appropriate molar ratio. Finally, the water soluble cluster **5** was prepared according to methods reported previously [32]. Complex **1** was characterized first by elemental analyses and spectroscopic methods. Crystals of complexes **1**–**3** are stable in air but were kept in darkness. Complexes **1–3 **were soluble in MeCN, CHCl_3_, CH_2_Cl_2_ DMSO, DMF and CH_3_OH, while complex **4** is slightly soluble in DMSO. Complex **5** is highly soluble in water, in DMSO and in DMF.

### 2.2. Vibrational Spectroscopy

The vibrational thioamide bands I and II, appear at 1496 and 1305 cm^−1^ in the IR spectra of complex **1** and lie at lower wavenumbers compared to the corresponding vibrational bands of the free ligands CMBZT, observed at 1504 and 1313 cm^−1^ [33]. Thioamide bands III-IV were observed at 1040–918 cm^−1^ in the spectra of the free ligand C–P and appear at 1026 and 905 cm^−1^, respectively, in the spectra of **1**. The bands at 1093 cm^−1^ in the IR spectra of **1** and **2–4** are assigned to the symmetric vibrations of the *ν*(C–P) bond [10]. Those at 515, 509 cm^−1^ **1,** 515, 509 cm^−1^ **2**, 516, 505 cm^−1^ **3** and 516, 505 cm^−1^ **4** are assigned to the antisymmetric vibrations of the *ν*(C–P) bond [10]. The corresponding *ν*(C–P) bands of the free tri-p-tolylphosphine ligand are found at 1089 cm^−1^ for the symmetric vibration and at 516 cm^−1^, 505 cm^−1^ for the anti-symmetric vibration. The bands at 169, 179, 125 and at 127 cm^−1^ in the Far-IR spectra of complexes **1–4** were assigned to the vibrations of the Ag–X bonds (X = Cl, I), respectively, [10]. The vibration at 248 cm^−1^ in the spectra of **1** is attributed to the Ag–S bonds [34].

### 2.3. Crystal and Molecular Structure of {[AgCl(CMBZT)(TPTP)_2_]*·*(MeOH)}  **(1)** and Structural Properties of {[AgCl(TPTP)_3_]*·*(0.5*·*H_2_O)} **(2)** and [AgI(TPTP)_3_] **(3)**

We have recently reported the crystal structure of the dehydrated complex [AgCl(TPTP)_3_] at 293 K [10]. The structure of {[AgCl(TPTP)_3_]*·*(0.5*·*H_2_O)} (**2**), described here, has been determined at 120 K. The structure of [AgI(TPTP)_3_] (**3**), determined at 120 K, is a new polymorph of the complex reported previously at 293 K and 140 K [10, 30]. Strong Ag–Ag interactions, due to quasiaromaticity calculated, were found in the structure of complex **4** re-determined at 120 K (With Ag–Ag Ǻ) (Figure 3).

**Figure 1:**
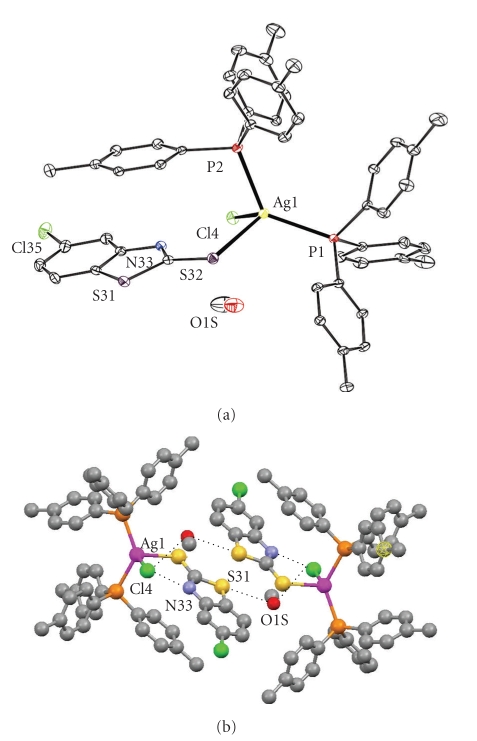
(a) ORTEP diagram of the crystal structure of complex **1**. (b) Intramolecular interactions (N33⋯Cl4, S31⋯O1S and O1S⋯Cl4) in **1**.

**Figure 2:**
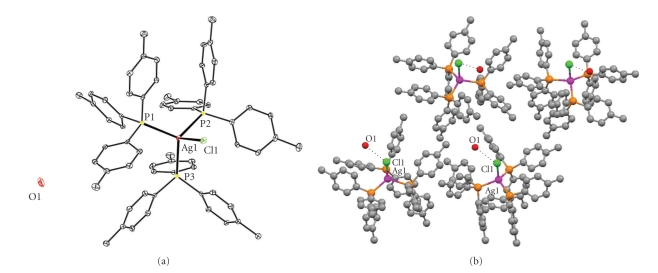
(a) ORTEP diagram of the crystal structure of complex **2**. (b) Packing diagram and hydrogen bonding interactions (Cl1⋯O1) in **2**.

 The crystal structures of complexes **1**-**2** are shown in Figures 1 and 2, while selected bond lengths and angles for **1–3** are given in Table 1 in comparison with those found in [10].

Two P atoms from the TPTP ligands, one S from CMBZT and one Cl atom form the tetrahedral arrangement around the Ag ion in complex **1**. The two Ag–P bond lengths are Ag1–P1 = 2.4357(18) Ǻ and Ag1–P2 = 2.5013(16) Ǻ which correspond closely with those found in [Ag(PPh_3_)(L)Br]_2_ (PPh_3_ = triphenylphosphine and L = 2 pyrimidine-2-thione) where Ag(1)-P(1) = 2.4390(7) Ǻ [34] and in [Ag_2_X_2_(l-S-pySH)_2_(PPh_3_)_2_] (X = Cl, Br and l-S-pySH = pyridine-2-thione) where the Ag–P bond distances are 2.435(1) Ǻ and 2.441(1) Ǻ, respectively, [35]. 

The Ag–S bond distance in **1** (Ag1-S32 = 2.6486(16) Ǻ) is longer than the corresponding bond distances reported for the terminal Ag-S bonds found in [Ag(PPh_3_)(L)Br]_2_ (PPh_3 _= triphnylphosphine and L = 2 pyrimidine-2-thione) (Ag(1)-S(1) = 2.5548(9) Ǻ [34]) and in [Ag_2_X_2_(*μ*-S-pySH)_2_(PPh_3_)_2_] (X = Cl, Br and *μ*-S-pySH = pyridine-2-thione) (Ag-S_terminal_ are 2.583(1) Ǻ and 2.608(1) Ǻ, resp., [35]). The Ag–S bond distance in **1** is closer to the bond length of bridging Ag-S bonds measured in [Ag_2_X_2_(*μ*-S-pySH)_2_(PPh_3_)_2_] (X = Cl, Br and *μ*-S-pySH = pyridine-2-thione) (Ag-S_bridging_ are 2.721(1) Ǻ and 2.631(1) Ǻ, resp., [35]). This might be due to intramolecular contact S31⋯O1S_(i) = 3.240(6) Ǻ (Figure 1(b)) (the symmetry transformation used to generate the equivalent atoms for (i) = −*x*, −*y*, 1 − *z*). 

The Ag1-Cl4 bond distance in **1** is 2.6736(13) Ǻ and is longer than the corresponding Ag-Cl bond distance found in [Ag_2_Cl_2_(*μ*-S-pySH)_2_(PPh_3_)_2_] (pySH = pyridine-2-thione) (Ag-Cl = 2.530(1) Ǻ [35]). This is due to the participation of the Cl atom in two strong hydrogen bonding interactions, one inter-molecular (N33⋯Cl4 = 3.083(4) Ǻ) and one intramolecular (O1S⋯Cl4 = 3.147(5) Ǻ) (Figure 1(b)). The hydrogen bonding interactions which involve the O atom of the methanol lead to the formation of a dimer (Figure 1(b)). 

 The bond angles around the Ag atom show variations from those in an ideal tetrahedron and range from P1-Ag1–P2 = 129.05(5)° to P2-Ag1-Cl4 = 95.13(5)° (see Table 1). 

The refinement of the absolute structure of **2 **showed that it consists of a mixture of enantiomers with ratio 0.81 :  0.19. Significant differences between the bond distances and angles of complex **2** at 120 K and the anhydrous form at 293 were observed and the values are also given in Table 1 for comparison. Although, the Ag–P bond lengths are shortened by decreasing the temperature (Table 1), the Ag-Cl bond distance remains unchanged (Table 1). This is attributed to the formation of a strong hydrogen bonding interaction involving the Cl atom in **2** (H1B[O1]⋯Cl1 = 2.42(10) Ǻ, O1-H1B⋯Cl1 = 150(18)°) (Figure 2(b)), in contrast to the case of the dehydrated complex [AgCl(TPTP)_3_], where no such bonds are formed. 

 The crystal structure of complex **3 **determined at 120 K (this work) is a new polymorphic form of the corresponding structures determined at 140 K and 293 K [10] (120 K: monoclinic in space group C2/c with *a* = 22.7429(10) Ǻ, *b* = 11.0093(3) Ǻ, *c* = 44.8281(18) Ǻ, *β* = 102.9780(10)°, and at 140 K. At 293 K: triclinic in space group P-1 with (140 K) *a* = 11.0058(5) Ǻ, *b* = 11.4509(5) Ǻ, *c* = 22.9459(8) Ǻ, *α* = 99.461(3)°, *β* = 91.648(3)°, *γ* = 106.350(4)° [10]. The general trend in complex **3** is the increasing of bond lengths; by increasing of temperature, for example the average Ag–P bond lengths in the various polymorphs are: at 120 K (new polymorph) 2.511 Ǻ, at 140 K (old polymorph) 2.537 Ǻ and at 293 K (old polymorph) 2.547 Ǻ. More important changes were observed in the bond angles between the three polymorphs (see Table 1). 

**Figure 3:**
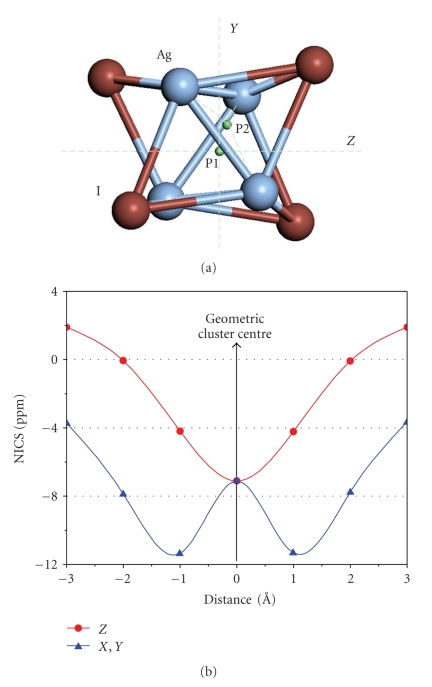
(a) The *C_2_* axes in the Ag_4_I_4_ core of the cluster **4,** along which NICS values have been calculated. (b) Plot of the NICS values calculated along the three *C_2_* axes.

Strong Ag–Ag interactions exist in the structure of complex **4**, consisted of four silver(I) ions bridged by *μ*_3_-iodide ions, forming a prismatic core (Figure 3) and are described here for the first time (both Ag–Ag = 3.1182(3) Å), with the Ag–Ag bond distances being shorter than the sum of their van der Waals radii (4.20–4.74 Å [36]) indicating a d^10^-d^10^ interaction. 

### 2.4. Computational Studies

A computational study utilizing the method of nucleus-independent chemical shifts (NICS) was employed for **4** in order to verify the Ag–Ag bonding interactions. Theoretical as well as experimental evidence of aromaticity in all-metal systems [20–29, 37–39] has attracted immense attention. This type of molecule contains either an aromatic cycle completely composed of metal atoms [40] or ligand-stabilized aromatic clusters with the ligands being either terminal or bridging adjacent metal atoms [41, 42]. In addition, aromatic clusters involving d-orbitals in pseudo-octahedral and in tetrahedral three-dimensional metal cages were comprehensively studied by the use of DFT methods [43]. In this work, we extend our study of aromaticity applied to laboratory data [10, 29] by means of magnetic criteria, using the method of nucleus-independent chemical shifts (NICS) proposed by Corminboeuf et al. in 1996 [21] to assess the aromatic character of cyclic structures. The calculated NICS indexes were obtained by calculating the negative isotropic value of the absolute NMR shieldings at the Ag_4_I_ 4_ cluster centre (P1) and at distances ranging from −3.0 to +3.0 Å in all three dimensions with a step of 1.0 Å resulting in 19 ghost atoms plus one ghost atom located at the centre (P2) of the plane delimited by the three nearest Ag atoms (NICS-3Ag) (Figure 3(a)). Significantly negative (i.e., magnetically shielded) NICS values (in ppm) indicate aromaticity while small (close to zero) NICS values represent non-aromaticity. 

 The variation of NMR shieldings along the three *C_2_* axes with the distance from the cluster barycentre is illustrated in Figure 3(b). The inner region is shielded up to −11.4 ppm at a distance of ±1 Å whereas NICS(0) is –7.1 ppm. The corresponding value for benzene, at the same level of theory, is −9.0 ppm. The results are indicative of diatropic regions attributed to the electron delocalization of d or (d-p) *π*-type orbital electron density between the four Ag and I atoms [10, 25, 26, 29, 44] which also justifies the equalization of the Ag–I bonds in the homometallic cluster. The symmetrical fluctuations of the NICS values as well as the similar ones obtained along *X* and *Y* axes are explained by the *S_4_* symmetry motif adopted by the Ag_4_I_4_ fragment. Lower NICS values are found along the *Z* axis due to the weak bonding interaction between the two Ag atoms sited at 3.539 Å apart; this distance is closer to the twice the van der Waals radius for silver (4.20–4.74 Å [36]). NICS-3Ag value is −7.3 ppm, consistent with the above results. This quasi aromaticity results in strong Ag–Ag interactions and in higher stability of the Ag_4_I_4_ core.

### 2.5. Biological Tests

#### 2.5.1. Cytotoxicity

Complexes **1–4** and the water soluble silver(I) cluster of formula {[(Et_3_NH)^+^]_2_ *·*[Ag_6_(*μ* *_3_-*Hmna)_4_(*μ* *_3_-*mna)_2_]^2-^ *·*(DMSO)_2_ *·*(H_2_O)} (**5**) (H_2_MNA = 2-mercaptonicotinic acid, C_6_H_5_NO_2_S) [32], were tested for their in vitro cytotoxic activity against leiomyosarcoma cancer cells (LMS) (mesenchymal tissue) from the Wistar rat, polycyclic aromatic hydrocarbons (PAH, benzo[a]pyrene) carcinogenesis. The cell viability and the cell growth proliferation activities were evaluated with Trypan Blue and Thiazolyl Blue Tetrazolium Bromide (MTT) assays, respectively. The IC_50_ values for cell viability are: 8 ± 0.39 *μ*M (**1**), 0.8 ± 0.08 *μ*M (**2**), 1.5 ± 0.06 *μ*M (**3**), 13.2 ± 0.42 *μ*M (**4**) and 4.5 ± 0.23 *μ*M (**5**). Thus, the order of the complexes activity towards cell viability is **2 **> **3 **> **5 **> **1 **> **4**, indicating that complex **2** is the strongest one. The IC_50_ values for cell growth proliferation are: 13.7 ± 3.06 *μ*M (**1**), 1.3 ± 0.2 *μ*M (**2**), 2.9 ± 0.31 *μ*M (**3**), 21.2 ± 1.93 *μ*M (**4**) and 40.3 ± 2.62 *μ*M (**5**). The order of the inhibitory activity of the complexes in cell growth proliferation is **2 **> **3 **> **1 **> **4 **> **5**. Thus, the chloride containing complex **2** showed the strongest activity against LMS cells. The IC_50_ value for cell viability determined for **2** is similar to the corresponding value found for the de-hydrated complex (0.7 *μ*M) [10]. However, the IC_50_ value for cell viability determined for **3** varies significantly (twofold) from the corresponding value found for its polymorphic form (0.8 *μ*M) [10]. The higher IC_50_ value in cell viability found for **4** (13.2 *μ*M) compared with the corresponding ones for **1–3** (0.8–8 *μ*M) might be attributed to the high stability of **4** due to the quasiaromaticity detected (see above).

The activities of complexes **2**, **3**, and **5,** against cells viability are comparable to that of cisplatin, with the corresponding (IC_50_) value for cisplatin against LMS cells being 4-5 *μ*M [12]. However, the inhibitory activities of complexes **1–4, **on the cells growth proliferation are higher than that of cisplatin, with IC_50_ value against LMS cells being 25 *μ*M. 

 It is noteworthy to mention that none of the complexes **1–5** showed any activity on cell growth proliferation of MRC-5 cells (normal human fetal lung fibroblast).

#### 2.5.2. Flow Cytometry

**Figure 4:**
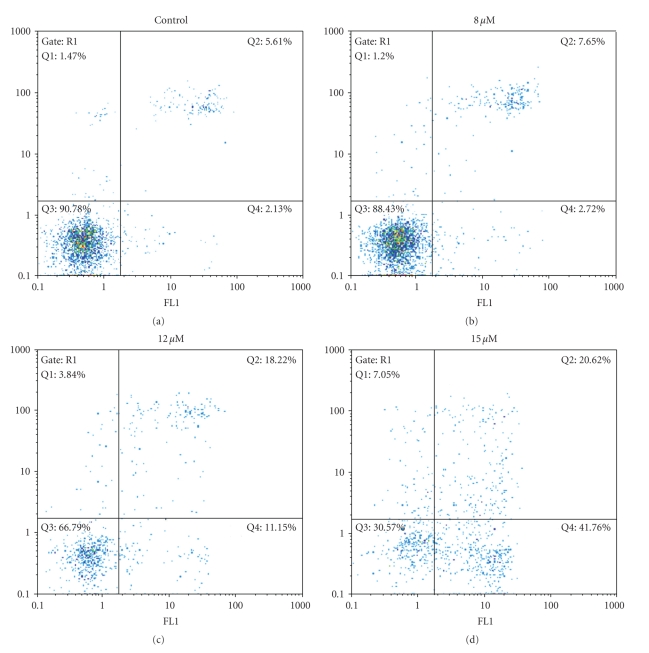
Flow cytometry assay summary results for LMS cells, treated with various concentrations of **1 **(8, 12, and 15 *μ*M) for 48 hours of incubation, in comparison with the untreated cells (control).

A flow cytometry assay was used to quantify apoptotic or necrotic cells treated with compound **1**. Treated and untreated LMS cells were stained with Annexin V-FITC and Propidium Iodide (PI). Figure 4, shows the dose-dependent cytotoxic response in LMS cells through apoptosis when treated with **1**. Compared to untreated LMS cells which showed a total of 9.22% of background cell death, the cells treated with complex **1** at 8 *μ*M showed 10.37% apoptosis (early apoptotic cells (Ann+/PI−) and late apoptotic cells (Ann+/PI+)) and 1.20% necrosis. When LMS cells were treated with 12 *μ*M of **1**, the cell death was increased to 29.37% apoptosis (early and late apoptotic cells) and 3.84% necrosis. At higher than the IC_50_ concentration of **1** (15 *μ*M), 62.38% of LMS cells were early and late apoptotic cells and 7.05% were necrotic. Thus, **1** causes a dose-dependent cytotoxic response in LMS cells through apoptosis. Although, no direct comparison can be made due to the different cell lines used, the apoptosis of HeLa cells induced by 33.75 *μ*M solution of organogold(III) complexes containing the “pincer” iminophosphorane ligand (2-C_6_H_4_-PPh_2_=NPh) of formula [Au{**κ** ^2^-C,N-C_6_H_4_(PPh_2_=N(C_6_H_5_)-2}Cl_2_] is 23.3% [45], in contrast to the 62.38% of **1 **against LMS cells, which might indicate stronger interaction of **1** with DNA.

#### 2.5.3. Anti-Inflammatory Activity

**Figure 5:**
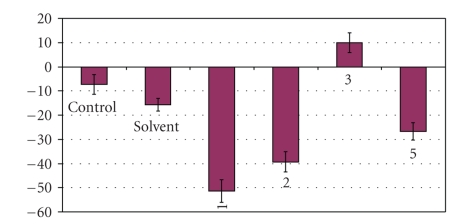
Change of the burnt surfaces after 21 days treatment with the solvent and complexes **1–3** and **5** in contrast to the burnt surface at 0 days.

Apoptotic cell death requires interaction of the complexes with DNA, which results into antimicrobial activity and to the burn wound recovery of the complexes [9]. Complexes **1–3** and **5** were tested for their anti-inflammatory activity. Figure 5 shows the changes of the burnt surface of animals after 21 days treatment with solvent (glyceryl trioctanoate) and complexes **1–3** and **5** in contrast to the burnt surface at 0 days, in comparison with the corresponding value for the untreated animals (control). The animals of the control group showed 7.38% decrease of the burnt surface, while the animals of the solvent group showed 15.67% decrease of the burnt surface. Furthermore, animals of the group treated with **1**, **2** and **5**, showed 51.33%, 39.32% and 26.76% decrease of the burnt surface, respectively. Therefore, the order of the anti-inflamatory activity caused by silver(I) complexes is **1 **> **2 **> **5 > **⋯**> 3**. Thus, the chloride containing complexes **1** and **2** were found to decrease the burnt surface most effectively. The mixed ligand Ag(I) with phosphine and the thione CMBZT, complex **1**, showed the strongest anti-burn activity which might be due to the synergistic effect of the chlorine and thioamide ligands. 

## 3. Conclusions

Silver(I) halide complex **1 **was synthesized and characterized by single crystal X-ray diffraction analysis. Complex **2** X-ray crystal structure on the other hand, showed that it deferred from the structure of complex [AgCl(TPTP)_3_] [10] containing a water molecule. The re-determination of the the structure of [AgI(TPTP)_3_] (**3**) determined at 120 K is a new polymorphic form of the complex reported previously at 293 K and 140 K [10]. A computational study using the method of nucleus-independent chemical shifts (NICS) showed that the Ag_4_I_4_ core of complex **4** exhibits strong quasiaromaticity, which at the inner region is shielded up to −11.4 ppm, resulting in the formation of strong Ag–Ag bonds. 

 The results of testing complexes **1–5**, for their in vitro cytotoxic activity against leiomyosarcoma cancer cells (LMS) (mesenchymal tissue) from the Wistar rat, polycyclic aromatic hydrocarbons (PAH, benzo[a]pyrene) carcinogenesis, showed that the chloride containing complex **2** had the strongest activity against LMS cells. The order of the complexes activity towards cell viability is **2 **> **3 **> **5 **> **1 **> **4**, while the order of the inhibitory activity of the complexes in cell growth proliferation is **2 **> **3 **> **1 **> **4 **> **5**. The IC_50_ value for cell viability determined for **2** is similar to the corresponding value found for the de-hydrated complex [10]. However, the IC_50_ value for cell viability determined for **3 (**crystallized in monoclinic, C2/c space group), varies significantly (twofold) from the corresponding value found for its polymorphic form (crystallized in triclinic, P-1 space group) [10]. The low biological activity of **4** might be attributed to its high stability due to the quasiaromaticity detected. The type of cell death in the case of complex **1 **was also evaluated by use of a flow cytometric assay. 

The results showed that **1** causes a dose-dependent cytotoxic response in LMS cells through apoptosis. Apoptotic cell death requires interaction of the complexes with DNA, which results into their antimicrobial activity and to the burn wound recovery [9]. 

 The chloride containing complexes **1** and **2** were also found to decrease the burnt surface most effectively. The order of the anti-inflamatory activity caused by silver(I) complexes is **1 **> **2 **> **5 > **⋯**>** **3**. The mixed ligand Ag(I) with phosphine and the thione CMBZT, complex **1, **showed the strongest anti-inflammatory activity which might be due to the synergistic effect of the chloride and thioamide ligands. Among complexes **1–3** and **5**, complex **2,** which showed the strongest activity against cells viability and the highest inhibitory activity against cells proliferation, also found to exhibit strong burn wood recovery activity.

## 4. Experimental

### 4.1. Materials and Instruments

All solvents used were of reagent grade. Silver(I) chloride and Silver(I) iodide were prepared by mixing aqueous solutions of AgNO_3_ with the appropriate amount of NaCl and NaI (Merck or Aldrich), respectively. The precipitates were filtered off and dried in darkness. Tri-p-tolyl-phosphine and 5-chloro-2-mercaptobenzothiazole (Fluka, Aldrich) were used with no further purification. Melting points were measured in open tubes with a Stuart Scientific apparatus and are uncorrected. IR spectra in the region of 4000–370 cm^−1^ were obtained from KBr discs, while far-IR spectra in the region of 400–30 cm^−1^ were obtained from polyethylene discs, with a Perkin-Elmer Spectrum GX FT-IR spectrophotometer.

### 4.2. Synthesis and Crystallization of {[AgCl(CMBZT)(TPTP)_2_]*·*(MeOH)}   **(1)**, {[AgCl(TPTP)_3_]*·*(0.5*·*H_2_O)}  **(2)**, [AgI(TPTP)_3_]   **(3)** and {[AgI(TPTP)]_4_}  **(4)**

Complex **1** was synthesized as follows: A solution of 0.202 g CMBZT (0.5 mmol) in 10 ml methanol was added to a suspension of 0.072 g AgCl (0.5 mmol) and 0.304 g TPTP (1 mmol) in 10 ml acetonitrile and the mixture was stirred for 1 hour at 50°C. The clear solution was filtered off and the filtrate was kept in darkness. After a few days a pale yellow powder was precipitated. Crystals of complex **1** suitable for X-ray crystallographic analysis were obtained from a toluene/chloroform solution. Complexes **2–4** were prepared by heading toluene solutions of the appropriate amounts of silver(I) halides with TPTP under reflux for 3 hours. The clear solutions were filtered off and the filtrates were kept in darkness. Colorless crystals of complexes **2–4** suitable for X-ray crystallographic analysis were grown from the filtrates.

1: Yield: 44%; m.p: 178–181°C; {[C_49_H_46_P_2_AgCl_2_NS_2_]*·*(CH_3_OH)} (MW = 985.7); elemental analysis: found C = 60.75%, H = 5.21%, N = 1.39%, S = 6.87%; calcd: C = 60.92%, H = 5.11%, N = 1.42%, S = 6.50%. MID-IR (cm^−1^) (KBr): 3013, 2915, 1496, 1305, 1093, 1026, 802, 658, 515, 509, Far-IR (cm^−1^) (polyethylene): 248, 169.
2: Yield: 80%; m.p: 199–205°C; {[C_63_H_63_P_3_AgCl]*·*(0.5 H_2_O)} (MW = 1065.4); elemental analysis: found C = 70.55%, H = 6.24%; calcd: C = 70.43%, H = 6.09%. MID-IR (cm^−1^) (KBr): 3013, 2911, 1093, 800, 516, 509, Far-IR (cm^−1^) (polyethylene): 179.
3: Yield: 83%; m.p: 145–152°C; {C_63_H_63_P_3_AgI} (MW = 1147.8); elemental analysis: found C = 65.30%, H = 5.87%; calcd: C = 65.92%, H = 5.53%. MID-IR (cm^−1^) (KBr): 3008, 2915, 1093, 802, 516, 505, Far-IR (cm^−1^) (polyethylene): 125.
4: Yield: 56%; m.p: 278–283°C; {C_84_H_84_P_4_Ag_4_I_4_} (MW = 2156.5); elemental analysis: found C = 46.28%, H = 3.88%; calcd: C = 46.79%, H = 3.92%. MID-IR (cm^−1^) (KBr): 3013, 2911, 1095, 802, 516, 505, Far-IR (cm^−1^) (polyethylene): 127.

### 4.3. X-Ray Structure Determination

X-ray diffraction data from the crystals of **1** were collected on a KUMA KM4CCD four-circle diffractometer [46] with a CCD detector, using graphite-monochromated MoK_*α*_ radiation (*λ* = 0.71073Å) at 293(2) K. Unit cell parameters were determined by a least-squares fit [47]. All data were corrected for Lorentz-polarization effects and absorption [47, 48]. The structure was solved by direct methods using SHELXS-97 [49] and refined by a full-matrix least-squares procedure on *F^2^* with SHELXL-97 [49]. All non-hydrogen atoms were refined anisotropically while hydrogen atoms were located at calculated positions and refined using a “riding model” with isotropic displacement parameters based on the equivalent isotropic displacement parameter (U_eq_) of the parent atom. 

 X-ray diffraction data from the crystals of **2 **and** 3** were collected on a Bruker APEXII CCD diffractometer and data from crystals of **4** were collected on a Bruker CCD diffractometer, both at the window of a Bruker FR591 rotating anode (*λ* = 0.71073 Å) at 120(2) K. The data collections were driven by COLLECT [50] and processed by DENZO [51]. Absorption corrections were applied using SADABS [52]. The structures of **2 **and **3** were solved using SHELXS-97 [49] while that of **4** was solved in SIR2004 [53] and all three structures were refined in SHELXL-97 [49]. All non-hydrogen atoms were refined anisotropically. In **2** there is a water molecule present in the structure at 50% occupancy for which the hydrogen atom positions were located in the electron density. The remaining hydrogen atoms of **2** and all those in **3** and **4 **were refined using a “riding model” in a similar manner to those in structure **1**. For structure **2** the refined absolute structure parameter was 0.19 (3), indicating that the structure consists of a mixture of enantiomers with ratio 0.81 : 0.19. Significant crystal data are given in Table 2. 

 Supplementary data are available from CCDC, 12 Union Road, Cambridge CB2 1EZ, UK, (e-mail: deposit@ccdc.cam.ac.uk), on request, quoting the deposition numbers CCDC 715884 (**1**), 717733 (**2**), 717734 (**3**) and 717735 (**4**), respectively.

### 4.4. Computational Details

Calculations based on the molecular geometry acquired via X-ray diffraction methods were carried out with the Gaussian03W program package [54]. Magnetic shielding tensors for a ghost atom placed at different interior and near exterior positions of the Ag_4_I_4_ cluster were computed using the gauge-independent atomic orbital (GIAO) DFT method [55–57] within the B3LYP level of theory. Nucleus independent chemical shift (NICS) values were estimated with the B3LYP function using the Los Alamos ECP plus double zeta (LANL2DZ) basis set for the Ag atoms and the 6-311G(d,p) for all the others. 

### 4.5. Biological Tests

#### 4.5.1. Trypan Blue Assay

A trypan blue dye exclusion assay was used to determine cell viability. A cell suspension was prepared using brief trypsinization (250 *μ*L of trypsin-incubation at 37°C, 95% O_2_, 5% CO_2_). An equal volume of PBS was added and the suspension was mixed with 500 *μ*L of 0.4% Trypan blue solution and left for 5 minutes at room temperature. The stained (dead) cells and the total cells per square of the cell chamber were counted using Neubauer cytometer after 24 hours of incubation with different concentrations of complexes **1–4 **and **5**.

#### 4.5.2. MTT Assay

Cell growth inhibition was analyzed using the 3-(4,5-dimethylthiazol-2-yl)-2,5-diphenyltetrazolium bromide (MTT) assay. LMS cells cultured on 96-well plates were washed with PBS. A solution containing different concentrations of complexes **1–4** and **5** was then added. After incubation for 24 hours, 50 *μ*l of MTT was added in each well from a stock solution (5 mg/ml), and incubated for an additional 4 hours. Blue formazans were eluted from cells by adding 50 *μ*l of DMSO under gentle shaking and absorbance was determined at 570 nm (subtract background absorbance measured at 690 nm) using a microplate spectrophotometer (Multiskan Spectrum, Therno Fisher Scientific, Waltham, USA).

#### 4.5.3. Flow Cytometry

LMS cells were seeded onto six-well plates at a density of 6 × 10^4^ cells per well and incubated for 24 hours before the experiment. Cells were washed with PBS, treated with media containing various concentrations of **1** in DMSO (8, 12 and 15 *μ*M) and incubated for 48 hours. Supernatants and cells collected were centrifuged and cell pellets were suspended in calcium buffer 1× at a rate 10^5^ cells/100 *μ*l. Cells were stained with Annexin (BD 556420) and Propidium Iodide (Sigma P4864) in a dark room for 15 minutes. DNA content was determined on a FACScan flow cytometer (Partec ML, Partec GmbH, Germany). Percentage of apoptotic, necrotic and decompensate cells were calculated over all viable cells (100%).

### 4.6. Anti-Inflammatory Activity

#### 4.6.1. Substances Preparation

The complexes **1–3 **and** 5** were dissolved in Glyceryl trioctanoate minimum 99% (from Sigma-T9126), to a concentration of 2 × 10^−4^ M and the solutions were kept at 4°C during the experiment.

#### 4.6.2. Animal Preparation

All animals were anaesthetized with 1 ml of anaesthetic cocktail (50 mgr/10 ml Midazolame, 50 mgr/10 ml Ketamine, Sodium Chloride 0.9% at a ratio 1.5/0.5/3) given into the peritoneal. After anaesthetization and 5 cm^2^ of skin removal, all animals were burnt for 5 seconds at a specific area by candescent steely stamp. This stamp had been put over a Bunsen lamp for at least 10 minutes. The average of the total burnt surface area represented 1.6% of the total body surface. The burnt area was cleaned by a sterilized dry paper and impressed on a clear paper by a permanent marker, in order to estimate the burnt surface area. A planimeter was also used to determine the burnt surface. 

 After burning, the animals were separated into 6 groups (6 animals in each group). The first group was the control group (CG) in which the 6 animals had no treatment. The second group was the solvent group (SG), in which all the animals were treated only with the glyceryl trioctanoate as solvent. The other 4 groups were the experiment groups (EG1-EG4), in which animals were treated with the solutions of the complexes in glyceryl trioctanoate. The treatment was performed by inducted 1.75 ml/day solutions over the burnt area. The treatment was continued for 20 days for all groups. At the 21st day the final burnt surface areas were estimated.

#### 4.6.3. Animals and Its Treatment

Female Wistar rats (36), aged 5 months and weighting almost 190 gr were reared in the laboratory in community cages at controlled room temperature (20 ± 2°C), with controlled lighting (12 h light/12 h dark). Standard Wistar rat diet and water *ad libitum* were used in all the experiments. 

 Experiments on animals were handled with human care in accordance with the National Institutes of Health guidelines and the European Union directive for the care and the use of laboratory animals (Greek presidential decree No. 160 1991).

## Figures and Tables

**Table 1:** Selected bond lengths (Ǻ) and angles (deg) for compounds **1–3** with e.s.d.'s in parentheses in comparison with those found in [10, 30].

**1 **at 293(2) **K**		**2** at 120 K		[AgCl(TPTP)_3_]		**3** at 120 K		[AgI(TPTP)_3_] (polymorph of **3**)		[AgI(TPTP)_3_] (polymorph of **3**)	
				at 293 K [10]				at 140 K [10]		at 293 K [10]	
Bond Distances (Å)		Bond Distances (Å)		Bond Distances (Å)		Bond Distances (Å)		Bond Distances (Å)		Bond Distances (Å)	
Ag1–P1	2.4357(18)	Ag1–P1	2.5159(13)	Ag1–P1	2.5566(12)	Ag1–P1	2.511(2)	Ag1–P1	2.5208(15)	Ag1–P1	2.5294(17)
Ag1–P2	2.5013(16)	Ag1–P2	2.5438(13)	Ag1–P2	2.5347(11)	Ag1–P2	2.518(2)	Ag1–P2	2.5453(15)	Ag1–P2	2.558(2)
Ag1-S32	2.6486(16)	Ag1-P3	2.5342(14)	Ag1-P3	2.5609(11)	Ag1-P3	2.504(2)	Ag1-P3	2.5444(13)	Ag1-P3	2.5529(17)
Ag1-Cl4	2.6736(13)	Ag1-Cl1	2.6109(13)	Ag1-Cl1	2.6186(17)	Ag1-I1	2.8359(8)	Ag1-I1	2.8736(6)	Ag1-I1	2.8655(9)
C32-S32	1.666(4)										
C32-N33	1.345(5)										
N33-H33	1.0323										

Bond Angles (°)		Bond Angles (°)		Bond Angles (°)		Bond Angles (°)		Bond Angles (°)		Bond Angles (°)	

P1-Ag1–P2	129.05(5)	P1-Ag1-P3	115.56(5)	P1-Ag1-P3	108.40(4)	P3-Ag1–P1	111.40(7)	I1-Ag1–P1	101.55(4)	I1-Ag1–P1	102.37(5)
P1-Ag1-S32	109.04(5)	P1-Ag1–P2	118.85(4)	P2-Ag1–P1	115.89(4)	P3-Ag1–P2	114.32(8)	I1-Ag1–P2	112.12(4)	I1-Ag1–P2	111.54(5)
P2-Ag1-S32	103.91(6)	P3-Ag1–P2	108.03(4)	P2-Ag1-P3	118.22(4)	P1-Ag1–P2	112.77(7)	I1-Ag1-P3	98.57(3)	I1-Ag1-P3	99.44(5)
P1-Ag1-Cl4	113.91(5)	P1-Ag1-Cl1	104.56(5)	P1-Ag1-Cl1	108.88(5)	P3-Ag1-I1	103.79(5)	P1-Ag1–P2	118.13(5)	P1-Ag1–P2	117.81(6)
P2-Ag1-Cl4	95.13(5)	P3-Ag1-Cl1	109.07(5)	P3-Ag1-Cl1	99.54(5)	P1-Ag1-I1	101.52(6)	P1-Ag1-P3	111.87(5)	P1-Ag1-P3	111.94(6)
S32-Ag1-Cl4	102.30(5)	P2-Ag1-Cl1	99.04(5)	P2-Ag1-Cl1	104.17(5)	P2-Ag1-I1	111.95(6)	P2-Ag1-P3	112.31(5)	P2-Ag1-P3	111.77(6)

**Table 2:** Crystal data and structure refinement details for complexes **1–4**.

	1; 293 K	2; 120 K	3; 120 K	4; 120 K
Empirical formula	C_50_H_50_AgCl_2_NOP_2_S_2_	C_63_H_64_AgClO_0.5_P_3_	C_63_H_63_AgIP_3_	C_84_H_84_Ag_4_I_4_P_4_
Fw	985.7	1065.4	1147.8	2156.5
Temperature (K)	293(2)	120(2)	120(2)	120(2)
Cryst. System	Triclinic	Orthorhombic	Monoclinic	Tetragonal
Space group	P1	Pna2_1_	C2/c	I4_1_/a
a, Å	11.226(4)	20.2646(4)	22.7429(10)	24.0727(2)
b, Å	13.659(5)	26.0074(5)	11.0093(3)	24.0727(2)
c, Å	18.227(7)	10.5020(2)	44.8281(18)	13.8592(2)
*α*, deg	98.97(3)	90	90	90
*β*, deg	98.08(3)	90	102.9780(10)	90
*γ*, deg	113.97(4)	90	90	90
V, Å^ 3^	2457.4(16)	5534.86(19)	10937.5(7)	8031.34(15)
*Z*	4	4	8	4
*ρ*_calcd_, g cm^−3^	1.332	1.279	1.394	1.783
*μ*, mm^−1^	0.7	0.5	1.1	2.6
R, wR2 [I > 2*σ*(I)],	0.0458, 0.1279	0.0598, 0.1073	0.0723, 0.2064	0.0299, 0.0773
